# The Pattern of Practice in the Management of Early Diabetic Kidney Disease

**DOI:** 10.7759/cureus.68584

**Published:** 2024-09-03

**Authors:** Abdullah H Almalki, Laila F Sadagah, Ahmad Makeen, Mohamed E Balla, Meshari Alzahrani, Faisal Kouther, Ahmed Aljuffri, Ashraf Marwan, Eman Kotbi, Sarah Dahlan, Turki Banamah, Muhammed Awais, Majed Alharthi

**Affiliations:** 1 Department of Medicine, Nephrology Section, King Abdulaziz Medical City, Ministry of National Guard, Jeddah, SAU; 2 College of Medicine, King Saud Bin Abdulaziz University for Health Sciences, Jeddah, SAU; 3 Department of Medicine, King Abdullah International Medical Research Center, Jeddah, SAU; 4 Department of Family Medicine, Al Salama Hospital, Jeddah, SAU; 5 Department of Obstetrics and Gynecology, King Abdulaziz Medical City, Ministry of National Guard, Jeddah, SAU; 6 Department of Surgery, Pediatric Surgery Section, King Abdulaziz Medical City, Ministry of National Guard, Jeddah, SAU

**Keywords:** angiotensin-converting enzyme inhibitors (acei), chronic kidney disease (ckd), diabetic screening, diabetic kidney disease (dkd), albuminuria reduction

## Abstract

Background and objectives

Diabetes is a leading cause of kidney failure in various regions worldwide. To detect renal disease in individuals with diabetes, screening typically involves evaluating the glomerular filtration rate and measuring albuminuria. Although there are established guidelines for these screenings, adherence to them varies. This study aims to examine the prevalence of albuminuria screening among adults with diabetes mellitus (DM) and to assess the different practices in managing these patients across primary and tertiary care settings.

Methods

This cross-sectional observational study involved adult patients with DM attending outpatient clinics in both primary and tertiary care settings. Patient data were gathered using a standardized form, excluding those with established chronic kidney disease (CKD) who were under nephrology care.

Results

The study included 1,010 patients, with 303 (30%) from primary care clinics and 707 (70%) from tertiary care clinics. The cohort comprised 582 (58%) females, with a median age of 62 years (IQR: 55-70), and approximately 990 (98%) had type 2 DM (T2DM). Annual albumin-to-creatinine ratio (ACR) screening was conducted for 498 out of 1,010 patients (49%) (95% confidence interval {CI}: 46%-52%). Screening compliance was notably higher in primary care settings compared to tertiary care clinics. Older patients (over 60 years) and those with hypertension or cardiac conditions were less likely to undergo screening. Among those screened, 185 of 498 patients (37%) (95% CI: 33%-41%) had abnormal albuminuria (ACR > 3).

Conclusion

Albuminuria is a significant indicator of progressing renal disease and cardiovascular risk. The annual screening rate for albuminuria in diabetic patients is inadequate. Primary care physicians show better adherence to screening guidelines compared to their tertiary care counterparts. Increasing physician awareness about the importance of screening could improve guideline compliance and mitigate the adverse effects of albuminuria.

## Introduction

Diabetes mellitus (DM) imposes a significant strain on healthcare systems, affecting both patient health and lifestyle. In Saudi Arabia, diabetes is widespread across various regions, primarily due to sedentary lifestyles [1]. Among the disorders that can damage the kidneys, diabetic nephropathy is the leading cause of end-stage renal disease (ESRD) and a frequent microvascular complication in our region [2]. Therefore, timely screening and early detection are vital for preventing progression to more severe stages.

Renal involvement in diabetic patients is assessed through estimated glomerular filtration rate (eGFR) and albuminuria testing. Albuminuria is associated with an increased risk of cardiovascular events, kidney disease progression, and mortality [3]. Despite global awareness, screening rates remain insufficient. Factors influencing albuminuria levels include blood pressure control, target glycosylated hemoglobin (A1c) levels, and preexisting comorbidities [4].

For decades, renin-angiotensin-aldosterone system inhibitors (RAASI) have been the standard therapy for reducing albuminuria and delaying progression to renal failure [5]. The rate of prescription is not universal and varies between healthcare professionals. Despite the notable recent evolution in the management of proteinuria in patients with chronic kidney disease (CKD), RAASI still represents the backbone of therapy.

This article aims to evaluate the rate of albuminuria screening among adult DM patients in primary and tertiary care clinics who have not previously been followed by nephrology. We will examine the prevalence of albuminuria, the factors affecting its levels, and the patterns of RAASI prescription.

## Materials and methods

Methods

This study utilized a cross-sectional observational design to investigate the rate of screening for albuminuria using urinary albumin-to-creatinine ratio (ACR) among adult patients with diabetes mellitus (DM) attending King Abdulaziz Medical City, Ministry of National Guard, Jeddah, in both primary care and tertiary care settings. The study covered a three-month period from June to August 2023, during which data were collected from patients visiting primary care, general internal medicine, and endocrine clinics. Patient charts were reviewed retrospectively over 18 months. A total of 1,010 patients were included in the study, with 303 (30%) from primary care and 707 (70%) from tertiary care clinics. The participants were selected based on their attendance at the respective clinics during the study period.

The inclusion criteria included all adult patients (>18 years) with a history of diabetes who had regular clinical visits at least every three months and recent laboratory test results. The patients followed by nephrology services for established chronic kidney disease (CKD) were excluded. The study received ethical approval from the Institutional Review Board (IRB) of King Abdullah International Medical Research Center (KAIMRC), IRB number SP16-208-J, prior to data collection.

Patient characteristics were collected using a standardized data collection form, including demographic information, medical history, and relevant clinical indicators. Data collection was performed by trained research assistants following a standardized protocol to ensure consistency.

In addition to estimating the rate of albuminuria screening, the study aimed to investigate factors associated with a higher tendency for screening, the prevalence of urine albumin among diabetic patients, and the relationship between abnormal eGFR and albuminuria. Moreover, the study examined the tendency toward RAASI prescription in these patients.

Statistical analysis

Descriptive statistics, including means, standard deviations, frequencies, and percentages, were used to summarize patient characteristics and demographic information. To assess the differences between the patients who were screened and those not screened for albuminuria, appropriate inferential statistical tests were employed, such as independent t-tests for continuous variables (e.g., age) and chi-square tests for categorical variables (e.g., gender and medical history). Additionally, logistic regression analysis was performed to examine the association between various patient characteristics and the albuminuria screening. All statistical analyses were performed using the SPSS 24 software (IBM SPSS Statistics, Armonk, NY), and the significance level was set at p < 0.05.

## Results

Study population

A total of 1,010 patients participated, with 582 (58%) being female. The median age was 62 (IQR: 55-70), and 990 (98%) had type 2 diabetes mellitus (T2DM). Of the participants, 309 (55%) had a body mass index (BMI) greater than 30, and 735 (73%) had hypertension (see Table 1). Uncontrolled DM was prevalent, with 223 (40%) having an A1c level above 8%. Renin-angiotensin-aldosterone system (RAAS) blockers were prescribed to 647 out of 1,010 patients (64%).

**Table 1:** Patients' baseline screening and demographic characteristics. Data are presented as n (%) unless indicated otherwise. *Significant P value SD, standard deviation; PHC, primary healthcare; GIM, general internal medicine; CAD, coronary artery disease; CHF, congestive heart failure; eGFR, estimated glomerular filtration rate; CKD-EPI, Chronic Kidney Disease Epidemiology Collaboration; RAAS, renin-angiotensin-aldosterone system; A1c, glycated hemoglobin; DM, diabetes mellitus

	All patients (n = 1,010)	Screened (n = 498)	Not screened (n = 512)	P value
Demographic characteristics				
Female gender	582 (58%)	282 (57%)	300 (59%)	0.527
Age (years), mean (SD)	62 (12)	60 (12)	64 (12)	<0.001*
Age > 60 years	601 (60%)	271 (54%)	330 (64%)	0.001*
Center				
PHC	303 (30%)	241 (48%)	62 (12%)	<0.001*
Endocrine	271 (27%)	129 (26%)	142 (28%)	
GIM	436 (43%)	128 (26%)	308 (60%)	
Medical comorbidities				
Body mass index, mean (SD) (n = 568)	31.2 (6.3)	31.3 (6.1)	31 (6.5)	0.669
Body mass index > 30	309 (55%)	137 (56%)	172 (54%)	0.685
Hypertension	735 (73%)	338 (68%)	397 (78%)	0.001*
Cardiac disease	143 (14%)	52 (10%)	91 (18%)	0.001*
CAD	97 (10%)	39 (8%)	58 (11%)	
CHF	23 (2.3%)	6 (1.2%)	17 (3.3%)	
Other cardiac	23 (2.3%)	7 (1.4%)	16 (3.1%)	
Screening for eGFR	1,007 (100%)	497 (100%)	510 (100%)	0.579
eGFR CKD-EPI, mL/minute, mean (SD) (n = 1,007)	88 (18)	90 (19)	87 (18)	0.01*
eGFR < 60 mL/minute	99 (10%)	48 (10%)	51 (10%)	0.855
Use of RAAS inhibitors	647 (64%)	309 (62%)	338 (66%)	0.189
A1c testing and control of DM				
A1c testing, annual	555 (55%)	252 (51%)	303 (59%)	0.006*
A1c, %, mean (SD) (n = 555)	7.9 (1.7)	7.8(1.6)	8.0 (1.8)	0.103
A1c < 8%	332 (60%)	155 (62%)	177 (58%)	0.459

Kidney disease among diabetic patients

Among those screened, abnormal albuminuria (ACR > 3) was found in 185 out of 498 patients (37%) (95% confidence interval {CI}: 33%-41%). An eGFR of <60 mL/minute was observed in 99 out of 1,007 patients (10%) (95% CI: 8%-12%), and hypertension was present in 735 out of 1,010 patients (73%) (95% CI: 70%-76%).

Screening and management practice

Annual screening for ACR was conducted in 498 out of 1,010 patients (49%) (95% CI: 46%-52%). Compliance with ACR screening was notably higher among patients visiting primary care settings compared to those in tertiary care clinics, with 241 patients (80%) in primary care versus 257 patients following in tertiary clinics (48% in endocrine clinics and 29% in general internal medicine clinics) (p < 0.001) (Table 2). Patients in general internal medicine clinics were less likely to undergo screening compared to those in endocrine and primary care clinics, and those in endocrine clinics were less likely to be screened than those in primary care. Older patients (over 60 years) and those with hypertension or cardiac conditions were less likely to be screened. Annual screening for serum creatinine was carried out for 1,007 out of 1,010 patients (100%).

**Table 2:** Analysis of screening for albuminuria among patients with diabetes (factors associated with screening). *Significant P value ACR, albumin-to-creatinine ratio; BMI, body mass index; eGFR, estimated glomerular filtration rate; OR, odds ratio; CI, confidence interval; AOR, adjusted odds ratio

Variables	Rate of ACR screening	Univariate analysis				Multivariate analysis			
		OR	95% CI		P value	AOR	95% CI		P value
Care setting									
Primary care (n = 303)	241 (80%)	1				1			
Tertiary care (n = 707)	257 (36%)	0.15	0.11	0.2	<0.001	0.11	0.07	0.17	<0.001*
Age									
<60 (n = 409)	227 (56%)	1				1			
>60 (n = 601)	271 (45%)	0.66	0.51	0.85	0.001	0.95	0.62	1.46	0.810
Gender									
Male (n = 428)	216 (50%)	1				1			
Female (n = 582)	282 (48%)	0.92	0.72	1.18	0.527	0.81	0.54	1.20	0.295
Obesity, BMI > 30									
No (n = 251)	107 (43%)	1				1			
Yes (n = 309)	137 (44%)	1.07	0.77	1.5	0.685	1.29	0.87	1.89	0.202
Hypertension									
No (n = 275)	160 (58%)	1				1			
Yes (n = 735)	338 (46%)	0.61	0.46	0.81	0.001	1.09	0.66	1.80	0.733
Cardiac disease									
No (n = 867)	446 (51%)	1				1			
Yes (n = 143)	52 (36%)	0.54	0.37	0.78	0.001	0.64	0.39	1.05	0.075
eGFR < 60 mL/minute									
No (n = 908)	449 (49%)	1				1			
Yes (n = 99)	48 (48%)	0.96	0.64	1.46	0.855	1.62	0.88	2.98	0.123

## Discussion

Type 2 diabetes mellitus (T2DM) stands as the foremost contributor to chronic kidney disease (CKD) on a global scale. A recent meta-analysis indicates a pooled prevalence of 16.4% in Saudi Arabia [6]. The concurrent manifestation of T2DM and CKD exacerbates the risk of renal failure, cardiovascular incidents, and elevated mortality rates. Comprehensive renal assessment in diabetic individuals encompasses the evaluation of the estimated glomerular filtration rate (eGFR) and the quantification of elevated urinary albumin-to-creatinine ratio (UACR). Although esteemed guidelines such as the Kidney Disease: Improving Global Outcome (KDIGO) and the American Diabetes Association (ADA) advocate for annual UACR screening in T2DM patients [7,8], real-world adherence to these recommendations remains inconsistent, often falling short of the desired practice [9].

Our investigation highlighted notable inconsistencies in UACR screening among T2DM patients over an 18-month observational period. While eGFR assessments were near-universally conducted, only half underwent UACR evaluations. Notably, primary healthcare centers exhibited a more rigorous approach to UACR screening compared to specialized tertiary facilities. The demographic composition of our study predominantly consisted of hypertensive individuals, with approximately 97 patients (10%) having a history of coronary artery diseases and CKD stage 3. Intriguingly, even though A1c levels are pivotal in monitoring T2DM control, a substantial fraction lacked such evaluations within the study period. Of the evaluated cohort, 223 patients (40%) presented A1c levels exceeding 8. Elevated UACR levels correlated significantly with hypertension, cardiovascular diseases, and uncontrolled A1c, yet no association emerged between UACR and eGFR in the early disease phase.

Albuminuria, as an independent entity, is intrinsically linked with heightened CKD progression and mortality risks [10]. Despite robust guideline endorsements emphasizing UACR assessment in T2DM, real-world data consistently underscore subpar screening frequencies. Our data estimate UACR screening in T2DM adults at approximately 49%, congruent with findings from the Awareness, Detection and Drug Therapy in Type 2 Diabetes and Chronic Kidney Disease (ADD-CKD) study and select US healthcare organizations [9-11]. Even more alarmingly, other reports have documented even more limited screenings, ranging from 35% to 43% [12-14].

Upon closer scrutiny of determinants influencing albuminuria screening, univariate analysis suggested a reduced likelihood of screening among older patients and those with cardiovascular or hypertensive complications. However, multivariate adjustments revealed the care setting as the primary determinant, with tertiary clinics (including endocrinology and internal medicine) being less proactive compared to primary care settings. Such observations resonate with findings from the TRIAD study [15]. However, in contrast to the TRIAD study, our data suggest an augmented awareness and implementation of UACR screening protocols among primary healthcare professionals. This conjecture is further supported by registries indicating an escalating UACR screening trend among primary care providers [16]. The comparatively diminished screening frequency in tertiary settings might be attributed to superior accessibility to primary care or the extensive comorbidity profile characteristic of tertiary care, which might overshadow the emphasis on proactive screening.

In our cohort, albuminuria prevalence was reported in 185/498 (37%), with only a minor fraction exhibiting severely elevated levels. Amplified albuminuria levels showed a trend associated with heightened blood pressure, cardiovascular history, and suboptimal diabetes management. These observations align with regional data and insights from the CKD Prognosis Consortium [12,17,18]. Multiple studies corroborate our findings, emphasizing the role of elevated blood pressure, heart complications, and uncontrolled A1c in diabetic nephropathy and albuminuria [12,19-21]. Other determinants of heightened UACR, as documented in diverse studies, include prolonged T2DM duration, hyperlipidemia, and advancing age.

The administration of renin-angiotensin-aldosterone system inhibitors (RAASI) offers potential benefits, including decelerated CKD progression, diminished albuminuria, and reduced cardiovascular incidents. Regrettably, there remains a significant treatment gap, particularly in patients with manifest indications. In our diabetic cohort with albuminuria or hypertension, a concerning 37 (20%) and 117 patients (16%) were not receiving RAASI therapy. Although these findings underscore a need for improved RAASI administration, they also hint at enhanced awareness compared to prior reports [22,23].

Undeniably, screening for renal disease in T2DM is paramount, considering its role as a primary causative agent of end-stage renal disease (ESRD) worldwide. Albuminuria serves as an early harbinger of renal dysfunction in diabetics, underscoring the imperative of systematic screening. Nevertheless, it is crucial to acknowledge that a notable subset of T2DM patients with renal deficits display standard UACR values yet compromised eGFR. In our sample, 99 patients (10%) had CKD stage 3, with 41 (42%) being non-albuminuric, highlighting the importance of concurrent eGFR and UACR assessments [24,25].

Limitations

Our study, while instrumental in revealing practice patterns in early diabetic kidney disease management, has inherent limitations. Firstly, it is a cross-sectional endeavor and might not encapsulate evolving clinical practices. Secondly, since the data stem from electronic health records, there exists a potential for underreporting, especially in instances where UACR assessments were conducted externally. Finally, it is essential to ponder if the observed practice patterns in our setting can be extrapolated universally, given diverse healthcare settings, resources, and protocols.

## Conclusions

The disparity in UACR screening frequency for T2DM patients, despite concrete guideline recommendations, is alarming. Our study unravels that primary care settings, paradoxically, display heightened adherence to UACR screening compared to specialized tertiary facilities. Albuminuria prevalence, which is reported in our study, is consistent with similar rates worldwide. Furthermore, the salient treatment gap in RAASI administration among eligible patients needs bridging. For holistic diabetic kidney disease management, consistent and comprehensive renal assessment, encompassing both eGFR and UACR, remains indispensable. Extensive efforts aiming to raise awareness among healthcare professionals are essential to mitigate the significant risk associated with a lack of screening and optimizing the overall health of diabetic patients.
